# STING-Dependent Type I IFN Production Inhibits Cell-Mediated Immunity to *Listeria monocytogenes*

**DOI:** 10.1371/journal.ppat.1003861

**Published:** 2014-01-02

**Authors:** Kristina A. Archer, Juliana Durack, Daniel A. Portnoy

**Affiliations:** 1 Department of Molecular and Cell Biology, University of California, Berkeley, Berkeley, California, United States of America; 2 School of Public Health, University of California, Berkeley, Berkeley, California, United States of America; University of Toronto, Canada

## Abstract

Infection with *Listeria monocytogenes* strains that enter the host cell cytosol leads to a robust cytotoxic T cell response resulting in long-lived cell-mediated immunity (CMI). Upon entry into the cytosol, *L. monocytogenes* secretes cyclic diadenosine monophosphate (c-di-AMP) which activates the innate immune sensor STING leading to the expression of IFN-β and co-regulated genes. In this study, we examined the role of STING in the development of protective CMI to *L. monocytogenes*. Mice deficient for STING or its downstream effector IRF3 restricted a secondary lethal challenge with *L. monocytogenes* and exhibited enhanced immunity that was MyD88-independent. Conversely, enhancing STING activation during immunization by co-administration of c-di-AMP or by infection with a *L. monocytogenes* mutant that secretes elevated levels of c-di-AMP resulted in decreased protective immunity that was largely dependent on the type I interferon receptor. These data suggest that *L. monocytogenes* activation of STING downregulates CMI by induction of type I interferon.

## Author Summary

Current vaccines are successful at generating neutralizing antibodies, however there is a pressing medical need to find adjuvants that yield long-lived memory T cells. Immunization with the bacterium *Listeria monocytogenes* induces a robust protective immune response mediated by cytotoxic lymphocytes that are efficient at killing infected cells upon reinfection. When *L. monocytogenes* enters a cell, it secretes the small molecule cyclic diadenosine monophosphate (c-di-AMP), which activates the host protein STING leading to a type I interferon response. In this study, we tested whether STING activation plays a role in the generation of cytotoxic lymphocytes and protective immunity using a mouse immunization model. We found that in the absence of STING signaling mice restricted bacterial growth and maintained higher numbers of cytotoxic lymphocytes upon reinfection, whereas mice immunized in the presence of elevated levels of c-di-AMP were less protected. These results suggest that the inflammation induced by a bacterial pathogen can be detrimental to the development of adaptive immunity, which could provide new insights into vaccine development.

## Introduction

Cell-mediated immunity (CMI) is a critical component for protection against intracellular pathogens. Upon infection, the innate immune response provides resistance and initiates the development of antigen-specific lymphocytes including cytotoxic CD8^+^ T cells, which ultimately kill host cells harboring pathogens [1]. The Gram-positive bacterium *Listeria monocytogenes* has been used for decades as a model organism to investigate the generation of CMI, as infection induces a robust effector and memory CD8^+^ T cell response that restricts bacterial growth following a lethal secondary challenge, resulting in long-lived sterilizing immunity [2]. Although it is generally agreed that activation of the innate immune system is critical for the initiation of adaptive immunity [3], the specific signaling pathways necessary to elicit a robust protective immune response to *L. monocytogenes* remain poorly understood.

*L. monocytogenes* is detected by multiple innate immune signaling pathways during infection [4]. Following engulfment by macrophages and dendritic cells, the bacteria reside within phagosomes where they are detected by Toll-Like Receptors (TLRs), resulting in the activation of MyD88-dependent response genes [5]. By secreting a pore-forming cytolysin, listerolysin O (LLO), *L. monocytogenes* escapes into the cytosol where it replicates and polymerizes actin to facilitate cell-to-cell spread [6]. *L. monocytogenes* is detected by several cytosolic innate immune pathways leading to a cytokine profile distinct from that of LLO-deficient bacteria, which are restricted to the phagosome [5], [7].

The primary cytosolic sensor of *L. monocytogenes* is STING (stimulator of interferon (IFN) genes, also known as MPYS, MITA and ERIS), an ER-localized transmembrane protein [8]. STING is activated by cyclic dinucleotides (CDNs) that are either produced by a pathogen or by an endogenous cyclic GMP-AMP synthase that is activated by DNA [9], [10]. Direct binding of CDNs to STING activates a downstream signaling cascade involving TBK1 and IRF3 [11], [12], [13]. In the case of *L. monocytogenes*, cyclic diadenosine monophosphate (c-di-AMP) is secreted through bacterial multi-drug efflux pumps, leading to STING activation and transcription of IFN-β and co-regulated genes [14], [15]. STING-deficient macrophages or mice are unable to produce IFN-β in response to *L. monocytogenes* infection indicating that STING is required for the type I IFN response to *L. monocytogenes* [16], [17].

Purified CDNs are immunostimulatory *in vitro* and *in vivo*. Murine and human dendritic cells exposed to cyclic diguanosine monophosphate (c-di-GMP) or c-di-AMP exhibit enhanced surface expression of costimulatory markers and T cell proliferation. Mice mount a significant antibody response following co-administration of protein antigens with c-di-GMP or c-di-AMP [13], [18], [19], [20]. CDNs also stimulate cellular immune responses. Antigen-stimulated splenocytes from mice immunized with β-ααgalactosidase in the presence of c-di-GMP or c-di-AMP proliferate and secrete cytokines [18], [19]. These data indicate that CDNs are sufficient to elicit a cell-mediated adaptive immune response.

Entry of *L. monocytogenes* into the host cytosol is necessary to generate secondary protective immunity, as phagosome-restricted heat-killed or LLO-deficient bacteria do not elicit functional cytotoxic T cells and long-term memory responses [21], [22], [23]. The attenuated ActA-deficient mutant strain, which escapes the phagosome but fails to polymerize actin and spread to neighboring cells, is fully immunogenic to mice [24]. Furthermore, MyD88-deficient mice, while highly susceptible to acute infection with virulent *L. monocytogenes*, are fully protected following secondary lethal challenge when immunized with the ActA-deficient mutant [25], [26], [27], [28]. These findings suggest that phagosomal detection of *L. monocytogenes* during immunization is not sufficient for the development of protective immunity.

STING activation induces an array of IRF3-dependent genes [5] as well as NF-κB and STAT6-dependent genes [29], [30]. Since LLO-deficient bacteria fail to enter the cytosol and induce STING-related genes [5], [7], we hypothesized that the detection of *L. monocytogenes* by STING is required for CMI. In this study, we tested whether STING signaling plays an important role in the generation of protective immunity to *L. monocytogenes*.

## Results

### The STING signaling pathway is not required for protective immunity to *L. monocytogenes*

In the model of protective immunity used in these studies, mice were immunized with an attenuated yet immunogenic strain of *L. monocytogenes* that lacks the *actA* and *inlB* genes (ActA^−^Lm) and challenged 30–38 days later with 2LD_50_ (2×10^5^ colony forming units (CFU)) of wild-type *L. monocytogenes* (WT Lm). Previous studies typically immunize mice with 0.1LD_50_ of *L. monocytogenes* (1×10^7^ CFU ActA^−^Lm for C57BL/6 mice) [21]. At this high immunization dose, bacterial burdens in subsequently challenged mice are below the limit of detection. In contrast, a lower immunization dose of 10^3^ CFU (∼0.00001LD_50_ for C57BL/6 mice) still generated significant immunity as compared to naïve mice, but did not induce saturating immunity and thus revealed differences that might be missed using higher doses (**Fig. S1A**).

**Figure 1:**
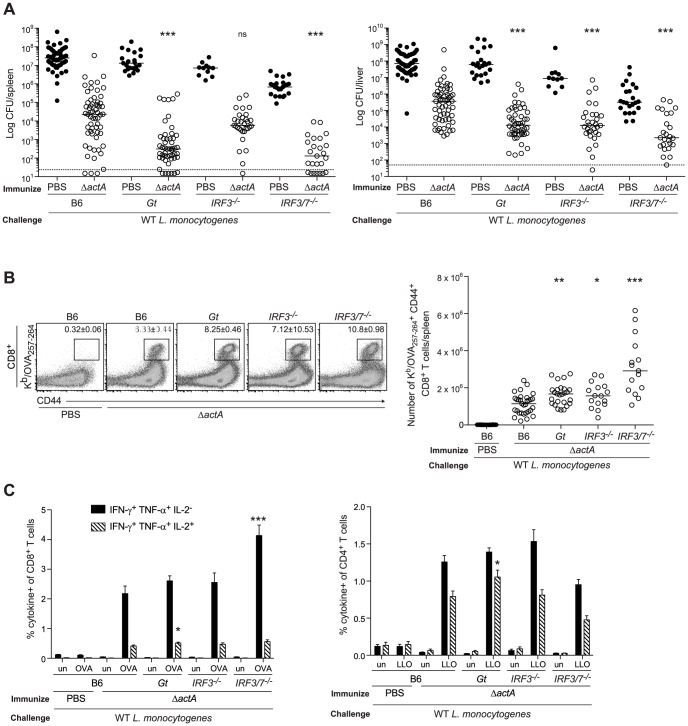
Mice lacking the STING signaling pathway generate a protective adaptive immune response following *L. monocytogenes* reinfection. B6, *Gt*, *IRF3^−/−^* and *IRF3/7^−/−^* mice were immunized intravenously with 10^3^ CFU ActA^−^Lm-OVA (Δ*actA*) (open circles) or administered PBS (closed circles) and 30–38 days later challenged with 2×10^5^ CFU WT Lm-OVA. Three days post challenge, **A**. bacterial CFU in the spleens and livers were enumerated. The dashed line represents the limit of detection. **B**. Splenocytes were stained with anti-mouse CD8, CD44 and K^b^/OVA_257–264_ tetramer and analyzed by flow cytometry. Quantitative analysis shows the total number of K^b^/OVA_257–264_ tetramer^+^ CD44^+^ CD8^+^ T cells/spleen. **C**. Splenocytes were either unstimulated (un) or stimulated with OVA_257–264_ (OVA) or LLO_190–201_ (LLO) peptides followed by intracellular staining with anti-mouse IFN-γ, TNF-α and IL-2. Quantitative analysis shows the percentage of IFN-γ^+^/TNF-α^+^/IL-2^−^ (solid bars) or IFN-γ^+^/TNF-α^+^/IL-2^+^ (lined bars) within the CD8^+^ or CD4^+^ population. Data are presented as the cumulative results from 5–12 (**A**), 3–6 (**B**) or 2–4 (**C**, mean ± SEM) independent experiments using at least five mice per group. Asterisks represent significance as compared to Δ*actA*-immunized B6 mice (*p<0.05, **p<0.005, ***p<0.0005).

To determine whether STING signaling is required for the generation of protective immunity to *L. monocytogenes*, mice lacking STING (*Goldenticket*, *Gt*) were immunized with 10^3^ CFU of ActA^−^Lm expressing ovalbumin (ActA^−^Lm-OVA) and challenged 30–38 days later with 2LD_50_s of WT Lm-OVA. Surprisingly, whereas naïve STING-deficient mice had similar bacterial burdens as naïve C57BL/6 (B6) mice, immunized STING-deficient mice had approximately 1.5–2 logs fewer bacteria in spleens and livers compared to immunized B6 mice (**Fig. 1A**). At higher immunization doses (10^4^ and 10^5^ CFU), the majority of B6 and STING-deficient mice had bacterial numbers below the limit of detection in the spleen and thus no significant differences could be observed (**Fig. S1B**). Since cytotoxic CD8^+^ T cells are the major mediator of *L. monocytogenes* clearance following secondary challenge [2], [31], the number of OVA-specific CD8^+^ T cells were measured by staining splenocytes with a MHC class I restricted OVA tetramer (K^b^/OVA_257–264_). STING-deficient mice had significantly higher total numbers of OVA-specific CD8^+^ T cells compared to B6 mice (**Fig. 1B**). These data suggested that STING-deficient mice exhibited enhanced immunity.

STING stimulation leads to the activation of the transcription factor, IRF3 [12]. In addition, IRF7 contributes to IFN-α production in response to *L. monocytogenes in vivo* [32]. To determine the role of these downstream effectors of STING signaling, mice lacking IRF3 (*IRF3^−/−^*) or IRF3 and IRF7 (*IRF3/7^−/−^*) were examined for protective immunity. Compared to B6 mice, IRF3-deficient mice had less bacteria in the livers and IRF3/7-deficient mice had less bacteria in the spleens and livers (**Fig. 1A**). Both groups had significantly higher numbers of OVA-specific CD8^+^ T cells than B6 mice (**Fig. 1B**), suggesting that these transcription factors contribute to the STING-mediated decrease in immunity.

To evaluate whether T cells from mice lacking STING signaling possessed effector functions, splenocytes from ActA^−^Lm-OVA-immunized mice were stimulated with either the MHC class I-restricted peptide OVA_257–264_, or the MHC class II-restricted peptide LLO_190–201_ from *L. monocytogenes* and measured for IFN-γ, TNF-α and IL-2 production by intracellular cytokine staining and flow cytometry. STING-deficient mice had significantly higher numbers of polyfunctional IFN-γ-, TNF-α- and IL-2- producing CD8^+^ and CD4^+^ T cells compared to B6 mice (**Fig. 1C**). IRF3/7-deficient but not IRF3-deficient mice also had higher numbers of IFN-γ- and TNF-α-producing CD8^+^ T cells. These data suggested that in the absence of STING or its downstream transcription factors IRF3 and IRF7, CD8^+^ T cell expansion, cytokine production and bacterial clearance was enhanced.

### In the absence of MyD88, STING plays a role in the innate but not the adaptive immune response

*L. monocytogenes* activates TLRs which signal via the adaptor MyD88 [4]. It is possible that both MyD88- and STING-dependent response genes play redundant roles in generating protective immunity to *L. monocytogenes*. To test this hypothesis, we bred mice lacking both MyD88 and STING (*MyD88^−/−^Gt*). Bone marrow-derived macrophages (BMMs) from MyD88/STING-deficient mice infected with WT Lm had low or non-detectable expression of the cytokines IFN-β, IL-12 p40, TNF-α and IL-6 (**Fig. S2A**).

To examine whether the loss of both the MyD88 and STING signaling pathways affect bacterial clearance during an acute infection, B6, MyD88- and MyD88/STING-deficient mice were infected with WT Lm. MyD88/STING-deficient mice had similar bacterial burdens as MyD88-deficient mice early after infection, however by day 3, had significantly higher CFU in the spleen and the liver (**Fig. S2B**). Although mice lacking MyD88 cannot survive infection with WT Lm, MyD88/STING-deficient mice died earlier than MyD88-deficient mice (**data not shown**). These data indicated that in the absence of MyD88, STING contributes to *L. monocytogenes* clearance during an acute response.

**Figure 2:**
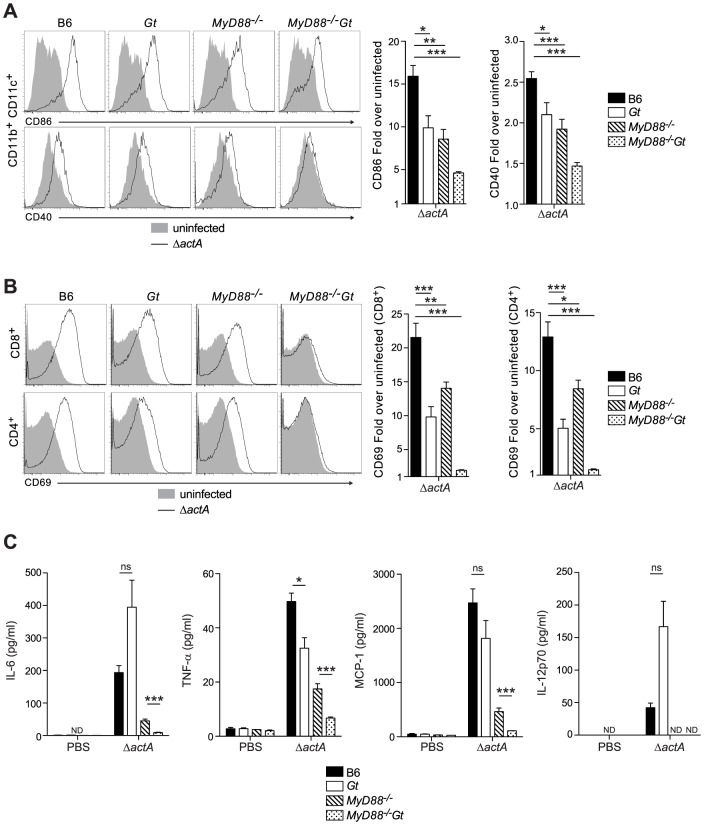
MyD88 and STING contribute to dendritic cell and T cell activation *in vivo*. B6 (closed bars), *Gt* (open bars), *MyD88^−/−^* (lined bars) and *MyD88^−/−^Gt* (dotted bars) mice were intravenously injected with either PBS (shaded histogram) or 10^5^ CFU ActA^−^Lm-OVA (Δ*actA*) (black line). One day post injection, **A**. splenocytes were isolated and stained with anti-mouse CD86, CD40, CD11b, CD11c or **B**. anti-mouse CD8, CD4 and CD69 and analyzed by flow cytometry. Quantitative analysis shows the fold increase of median fluorescence intensity over uninfected mice. **C**. Serum was measured for IL-6 and TNF-α, MCP-1 and IL-12p70 by Cytometric Bead Array. Data represent the mean ± SEM from 3 independent experiments with 3 mice per group (ns = not significant, *p<0.05, **p<0.005, ***p<0.0005).

To determine whether the loss of MyD88 and STING affects initiation of the adaptive response to *L. monocytogenes*, the upregulation of costimulatory molecules on splenic dendritic cells from immunized B6, STING-, MyD88- and MyD88/STING-deficient mice was measured. Surface expression of CD86 and CD40 was decreased in MyD88- and STING-deficient mice and further reduced in the MyD88/STING-deficient mice, suggesting an additive effect of these two pathways (**Fig. 2A**). Upregulation of the activation marker CD69 on CD8^+^ and CD4^+^ T cells was also decreased in both MyD88- and STING-deficient mice and ablated in MyD88/STING-deficient mice (**Fig. 2B**). Furthermore, IL-6, TNF-α and MCP-1 in the serum were mostly dependent on MyD88 but further reduced in the MyD88/STING-deficient mice. IL-12 p70 was non-detectable in either MyD88- or MyD88/STING-deficient mice (**Fig. 2C**). These data indicated that mice lacking MyD88 and STING signaling had significantly reduced dendritic and T cell activation and cytokine production *in vivo* in response to *L. monocytogenes* immunization.

**Figure 3:**
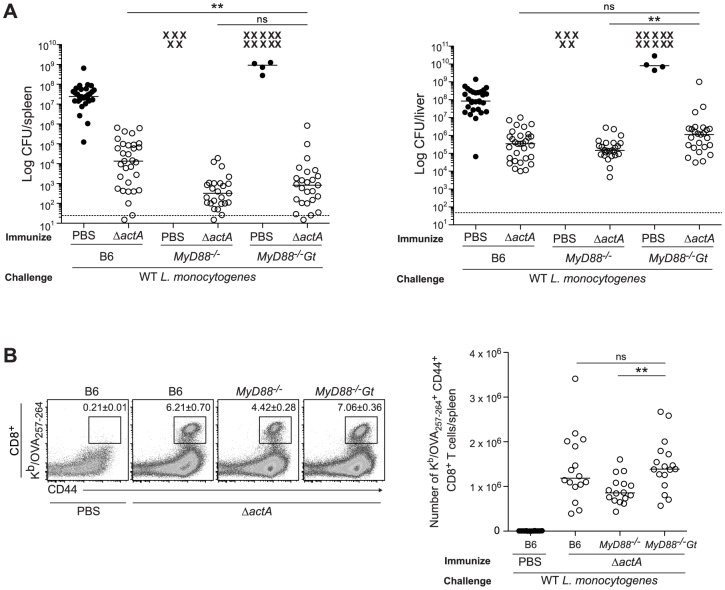
Mice lacking MyD88 and STING are protected from secondary challenge. B6, *MyD88^−/−^* and *MyD88^−/−^Gt* mice were immunized intravenously with 10^3^ CFU ActA^−^Lm-OVA (Δ*actA*) (open circles) or administered PBS (closed circles) and 30–38 days following immunization, challenged with 2×10^5^ CFU WT Lm-OVA. Three days post challenge, **A**. bacterial numbers in the spleens and livers were enumerated. An **X** marks each mouse that succumbed to infection prior to the conclusion of experiment. The dashed line represents the limit of detection. **B**. Splenocytes were stained with anti-mouse CD8, CD44 and K^b^/OVA_257–264_ tetramer and analyzed by flow cytometry. Quantitative analysis shows the total number of K^b^/OVA_257–264_ tetramer^+^ CD44^+^ CD8^+^ T cells/spleen. Data are presented as the cumulative results from 4–6 (**A**) or 3 (**B**) independent experiments (ns = not significant, *p<0.05, **p<0.005, ***p<0.0005).

Mice lacking both MyD88 and STING were tested for the ability to develop protective immunity. Similar to B6 and MyD88-deficient mice, MyD88/STING-deficient mice were protected following secondary lethal challenge and showed no signs of disease unlike naïve mice which were either moribund or dead at the time of sacrifice. MyD88/STING-deficient mice had a small increase in the number of bacteria in the livers compared to MyD88-deficient mice (**Fig. 3A**). However, MyD88/STING-deficient mice had significantly higher number of OVA-specific CD8^+^ T cells compared to MyD88-deficient mice (**Fig. 3B**), suggesting that the higher bacterial loads in the liver was likely due to loss of innate rather than adaptive immune responses. These data indicated that regardless of the presence or absence of MyD88, protective immunity to *L. monocytogenes* is enhanced in the absence of STING.

### Enhanced STING activation blocks the development of adaptive immunity

**Figure 4:**
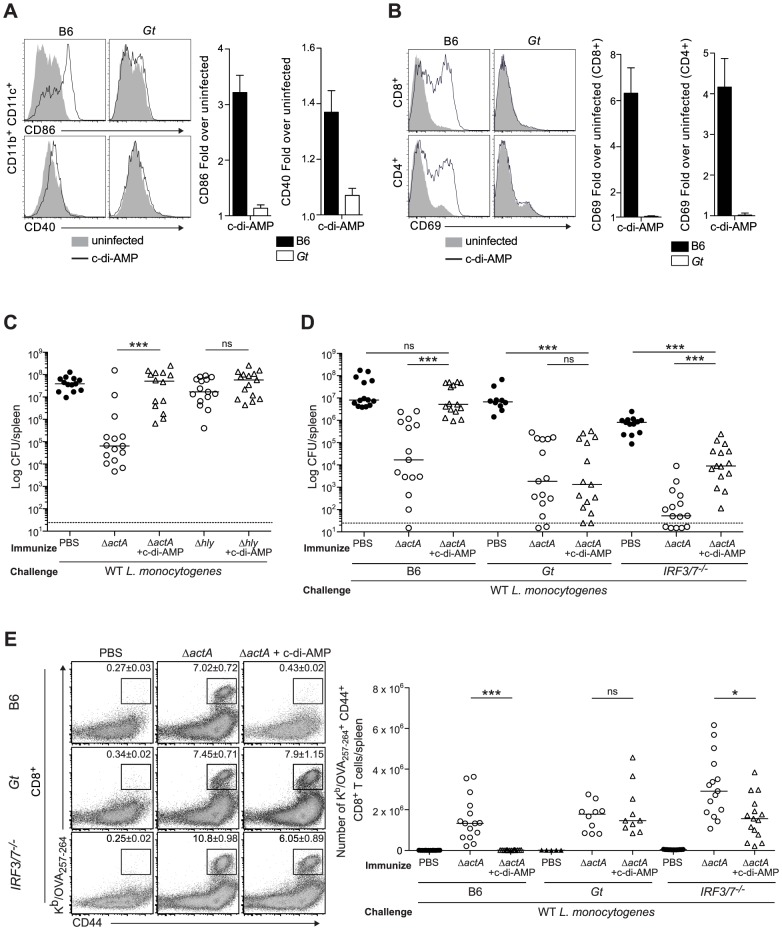
Administration of c-di-AMP during immunization inhibits CD8^+^ T cell expansion and protective immunity upon *L. monocytogenes* reinfection. **A** and **B**. B6 or *Gt* mice were intravenously injected with either 50 µg c-di-AMP (black line) or PBS (shaded histogram) and splenocytes were isolated 24 hours were stained with either **A**. anti-mouse CD86 and CD40 or **B**. anti-mouse CD8, CD4 and CD69 and analyzed by flow cytometry. Data are quantified as the fold increase of median fluorescence intensity over uninfected mice and presented as the mean ± SEM from 3 independent experiments using 3 mice per group (ND = not detectable). **C**. B6 mice were immunized intravenously with either 10^3^ ActA^−^Lm-OVA (Δ*actA*) or 10^4^ LLO^−^Lm-OVA (Δ*hly*) in the presence (open triangles) or absence (open circles) of 50 or 100 µg c-di-AMP, or **D**. B6, *Gt* or *IRF3/7^−/−^* mice were immunized with either 10^3^ CFU Δ*actA* in the presence (open triangles) or absence (open circles) of 100 µg c-di-AMP. Naive controls were administered sterile PBS (closed circles). Mice were challenged 30–38 days post immunization with 2×10^5^ CFU WT Lm-OVA and 3 days later CFU were enumerated in spleens. The dashed line represents limit of detection. **E**. Splenocytes isolated from **D** were stained with anti-mouse CD8, CD44 and K^b^/OVA_257–264_ tetramer and analyzed by flow cytometry. Quantitative analysis shows the total number of K^b^/OVA_257–264_ tetramer^+^ CD44^+^ CD8^+^ T cells/spleen. Data are presented as the cumulative results from 3 (**C** and **D**) or 2–3 (**E**) independent experiments (ns = not significant, *p<0.05, **p<0.005, ***p<0.0005).

Mice immunized with LLO-deficient *L. monocytogenes* fail to develop protective immunity [21], [22]. Since LLO-deficient bacteria do not activate STING, we hypothesized that c-di-AMP-mediated STING activation might be sufficient to restore immunity in LLO^−^Lm-immunized mice. In support of previous reports, we found that BMDCs stimulated with c-di-AMP secreted cytokines and upregulated costimulatory markers *in vitro* (**Fig. S3**). Furthermore, mice administered c-di-AMP intravenously exhibited increased surface expression of CD86 and CD40 on splenic dendritic cells and of the activation marker CD69 on splenic CD8^+^ and CD4^+^ T cells in a STING-dependent manner (**Fig. 4A and 4B**), indicating that c-di-AMP can induce an inflammatory response in our model.

To test our hypothesis, B6 mice were immunized with LLO^−^Lm-OVA in the presence of c-di-AMP. While ActA^−^Lm-OVA-immunized mice restricted bacterial growth following challenge, the co-administration of c-di-AMP with LLO^−^Lm-OVA did not rescue protective immunity (**Fig. 4C**). Instead, the presence of c-di-AMP significantly reduced immunity in ActA^−^Lm-OVA-immunized mice suggesting that STING signaling inhibits protective immunity to *L. monocytogenes* infection.

To further examine the effect of c-di-AMP on immunity to *L. monocytogenes*, B6, STING- and IRF3/7-deficient mice were immunized with ActA^−^Lm-OVA in the presence or absence of c-di-AMP. Following challenge, B6 mice immunized in the presence of c-di-AMP had significantly higher bacterial numbers and had fewer numbers of total and OVA-specific CD8^+^ T cells in the spleen than mice immunized with ActA^−^Lm-OVA alone (**Fig. 4D, 4E** **and S4A**). STING-deficient mice were protected and had a robust CD8^+^ T cell expansion confirming that c-di-AMP-mediated inhibition of immunity was STING-dependent. IRF3/7-deficient mice were significantly protected as compared to naïve mice and had a population of OVA-specific CD8^+^ T cells, suggesting that c-di-AMP-mediated inhibition is partially due to IRF3 and IRF7 (**Fig. 4D and 4E**). Interestingly, IRF3/7-deficient mice were less protected compared to mice immunized with ActA^−^Lm-OVA alone, indicating that STING-dependent, IRF3/7-independent signaling also plays a role in loss of protective immunity.

**Figure 5:**
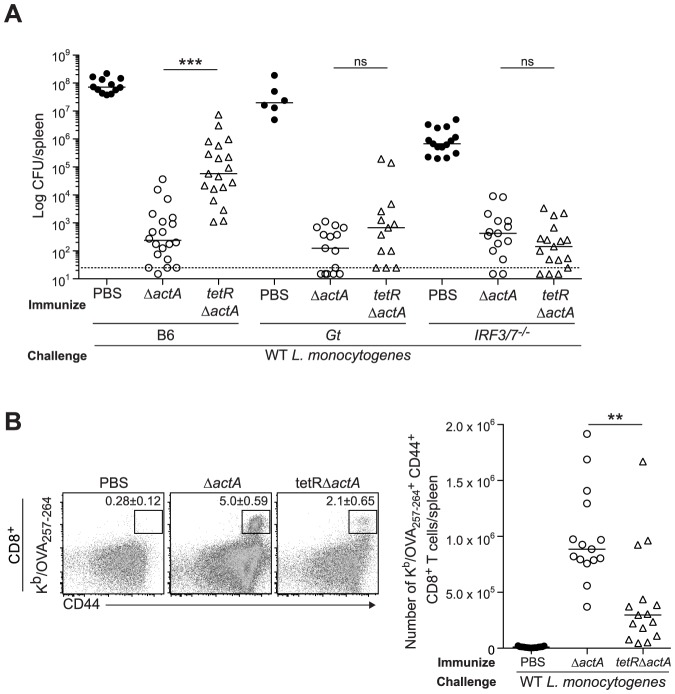
Immunization with a c-di-AMP hyper-secreting strain of *L. monocytogenes* reduces CD8^+^ T cell expansion and protective immunity following challenge. B6, *Gt* or *IRF3/7^−/−^* mice were immunized with either 10^3^ CFU Δ*actA* (open circles) or *tetR*ActA^−^Lm-OVA (*tetR*Δ*actA*) (open triangles). Naive controls were administered sterile PBS (closed circles). Mice were challenged 30–38 days post immunization with 2×10^5^ CFU WT Lm-OVA and 3 days later **A**. CFU were enumerated in spleens. The dashed line represents limit of detection. **B**. Splenocytes were stained with anti-mouse CD8, CD44 and K^b^/OVA_257–264_ tetramer and analyzed by flow cytometry. Quantitative analysis shows the total number of K^b^/OVA_257–264_ tetramer^+^ CD44^+^ CD8^+^ T cells/spleen. Data are presented as the cumulative results from 3–4 independent experiments (ns = not significant, *p<0.05, **p<0.005, ***p<0.0005).

Next, we evaluated a *L. monocytogenes* mutant, *tetR*::Tn917, that secretes 20-fold more c-di-AMP than WT *L. monocytogenes* [14], [15]. B6, STING- and IRF3/7-deficient mice were immunized with either ActA^−^Lm-OVA or the *tetR*::Tn*917* mutant in the ActA^−^Lm-OVA background (*tetR*ActA^−^Lm-OVA). B6 mice immunized with *tetR*ActA^−^Lm-OVA had significantly higher bacterial numbers in the spleen compared to ActA^−^Lm-OVA-immunized mice whereas STING- and IRF3/7-deficient mice exhibited no significant difference (**Fig. 5A**). Furthermore, B6 mice immunized with *tetR*ActA^−^Lm-OVA had a smaller population of total and OVA-specific CD8^+^ T cells compared to ActA^−^Lm-OVA-immunized mice (**Fig. 5B** **and S4B**). These data supported our finding that enhanced STING signaling lead to a reduction in protective immunity.

### Enhanced STING activation inhibits CD8^+^ T cell priming

**Figure 6:**
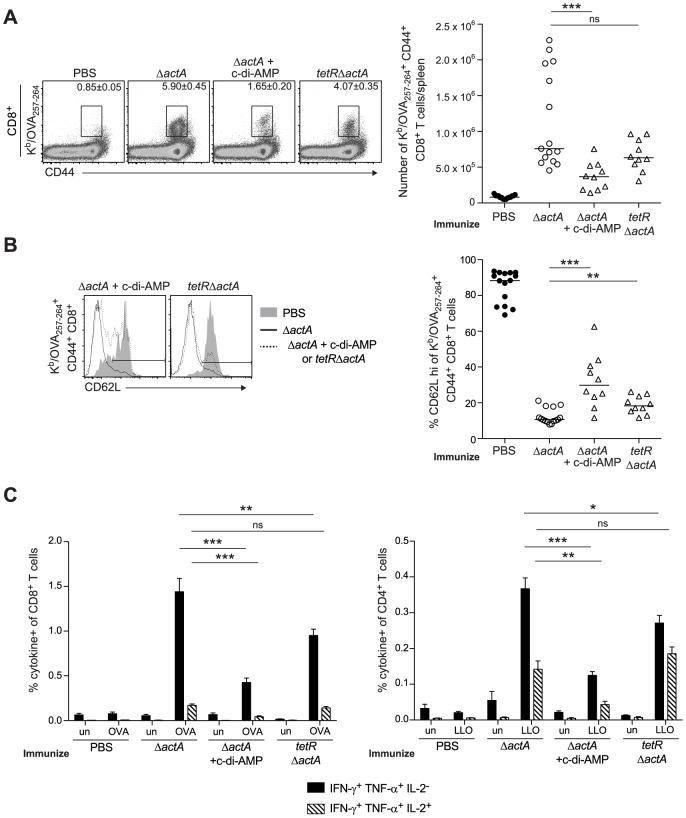
T cell priming is inhibited in the presence of enhanced STING activation. B6 mice were immunized with either 10^3^ CFU ActA^−^Lm-OVA (Δ*actA*) (open circles), Δ*actA* in the presence of 50 µg c-di-AMP or *tetR*ActA^−^Lm-OVA (*tetR*Δ*actA*) (open triangles). Naive controls were administered sterile PBS (closed circles). Splenocytes were isolated 7 days post immunization and stained with anti-mouse CD8, CD44, CD62L and K^b^/OVA_257–264_ tetramer and analyzed by flow cytometry. **A**. Quantitative analysis shows the total number of K^b^/OVA_257–264_ tetramer^+^ CD44^+^ CD8^+^ T cells/spleen. **B**. Histograms represent mice administered PBS (shaded), Δ*actA* (solid line) or Δ*actA* in the presence of c-di-AMP or *tetR*Δ*actA* (dotted line). Quantitative analysis shows the percentage of CD62L high-expressing cells of the CD8^+^ CD44^+^ K^b^/OVA_257–264_ tetramer^+^ population. **C**. Splenocytes were either unstimulated (un) or stimulated with OVA_257–264_ (OVA) or LLO_190–201_ (LLO) peptides followed by intracellular staining for anti-mouse IFN-γ, TNF-α and IL-2. Quantitative analysis shows the percentage of IFN-γ^+^/TNF-α^+^/IL-2^−^ (solid bars) or IFN-γ^+^/TNF-α^+^/IL-2^+^ (lined bars) within the CD8^+^ or CD4^+^ population. Data are presented as the cumulative results from 2–3 independent experiments (ns = not significant, *p<0.05, **p<0.005, ***p<0.0005).

To determine whether enhanced STING signaling reduces T cell priming, OVA-specific CD8^+^ T cells were measured at the peak of the primary response from mice immunized with ActA^−^Lm-OVA in the presence or absence of c-di-AMP or *tetR*ActA^−^Lm-OVA. At 7 days post immunization, mice immunized in the presence of c-di-AMP had significantly fewer OVA-specific CD8^+^ T cells compared to mice immunized with ActA^−^Lm-OVA alone. Mice immunized with the *tetR*ActA^−^Lm-OVA mutant also had a small decrease in antigen-specific cells (**Fig. 6A**). Furthermore, the small population of OVA-specific CD8^+^ T cells that were present in mice immunized with elevated STING activation had significantly higher surface expression of the naïve T cell maker CD62L, suggesting that enhanced STING signaling elicited fewer antigen-specific effector T cells (**Fig. 6B**). In addition, peptide-stimulated CD8^+^ and CD4^+^ splenocytes from ActA^−^Lm-OVA-immunized mice in the presence of c-di-AMP or *tetR*ActA^−^Lm-OVA-immunized mice produced fewer cytokines than those from ActA^−^Lm-OVA-immunized mice (**Fig. 6C**). These data indicate that mice immunized in the presence of enhanced STING signaling exhibited reduced T cell priming.

### STING-mediated loss of immunity is dependent on type I IFNs

**Figure 7:**
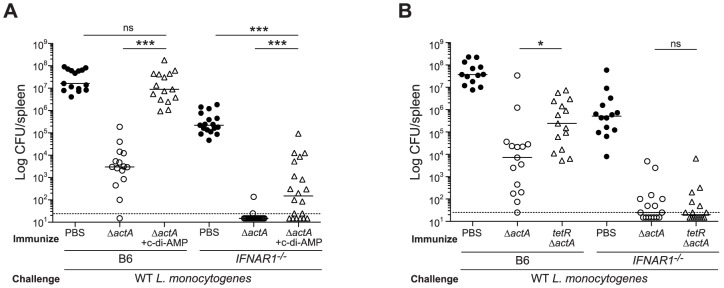
Mice lacking type I IFNs are partially rescued from c-di-AMP-mediated immune inhibition. **A**. B6 or *IFNAR1^−/−^* mice were immunized with 10^3^ CFU ActA^−^Lm-OVA (Δ*actA*) in the presence (open triangles) or absence (open circles) of 100 µg c-di-AMP or **B**. *tetR*ActA^−^Lm-OVA (*tetR*Δ*actA*) (open triangles). Naïve mice were administered PBS (closed circles). Mice were challenged 30–38 days later with 2×10^5^ CFU WT Lm-OVA and 3 days post challenge, CFU were enumerated from spleens. The dashed line represents limit of detection. Data are presented as the cumulative results from 3 independent experiments (ns = not significant, *p<0.05, **p<0.005, ***p<0.0005).

To determine the role of type I IFNs in STING-mediated CMI, IFN-αβR-deficient mice were tested for protective immunity. IFN-αβR-deficient mice restricted bacterial growth better than B6 mice (**Fig. 7A and 7B**), indicating that like STING- and IRF3/7-deficient mice, IFN-αβR-deficient mice also hyper-immunize. Although IFN-αβR-deficient mice immunized in the presence of c-di-AMP had higher bacterial numbers compared to mice immunized with ActA^−^Lm-OVA alone, these mice were significantly more protected than naïve mice, indicating a role for both type I IFN-dependent and independent mechanisms of suppression (**Fig. 7A**). IFN-αβR-deficient mice immunized with *tetR*ActA^−^Lm-OVA were completely protected (**Fig. 7B**). These data indicated that the c-di-AMP-mediated inhibition of protective immunity is largely dependent on type I IFNs. Interestingly, we found that mice immunized in the presence of the synthetic double-stranded RNA, polyinosinic∶polycytidylic acid (poly(I∶C)), a STING-independent agonist of TLR3 and IRF3, also lost the ability to restrict bacterial growth following challenge (**Fig S5**), suggesting that type I IFN-mediated inhibition of immunity is unlikely STING specific.

**Figure 8:**
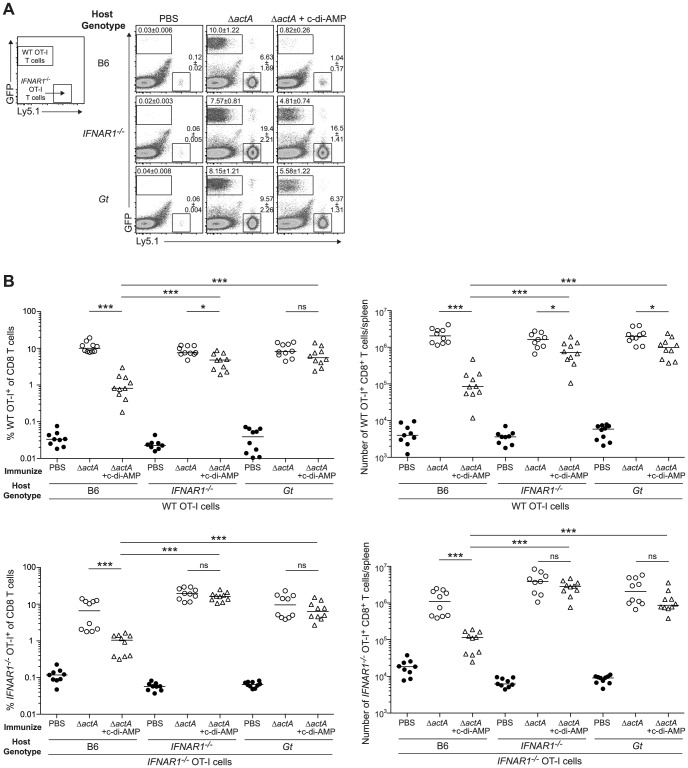
Type I IFN-mediated immune suppression is T cell extrinsic. **A**. B6, *IFNAR1^−/−^* and *Gt* mice were injected with 1∶1 ratio WT *GFP^+^ Ly5.2^+^* and *IFNAR1^−/−^ Ly5.1^+^* OT-I cells 1 day prior to immunization with ActA^−^Lm-OVA (Δ*actA*) in the presence (open triangles) or absence (open circles) of 50 µg c-di-AMP or administered PBS (closed circles). Splenocytes were isolated 7 days later and stained with anti-mouse CD8 and CD45.1 and analyzed by flow cytometry. Values in representative FACs plots show the median percentage of WT or *IFNAR1^−/−^* OT-I cells ± SEM within the CD8^+^ T cell population. **B**. Quantitative analysis shows the percentage (left panels) within the CD8^+^ T cell population or the total number of CD8^+^ T cells/spleen (right panels) of WT OT-I T cells (top panels) and *IFNAR1^−/−^* OT-I T cells (bottom panels). Data are presented as the cumulative results from 2 of 3 independent experiments (ns = not significant, *p<0.05, **p<0.005, ***p<0.0005).

We next determined whether inhibition of T cell priming by STING-dependent type I IFNs acted directly on lymphocytes. CD8^+^ T cells lacking the IFN-αβR undergo clonal expansion in response to primary *L. monocytogenes* infection so an adoptive transfer model could be used [33], [34]. B6, STING-, or IFN-αβR-deficient mice were injected with WT and IFN-αβR-deficient OT-I splenocytes and subsequently immunized with ActA^−^Lm-OVA in the presence or absence of c-di-AMP. At 7 days, WT and IFN-αβR-deficient OT-I cells expanded in ActA^−^Lm-OVA-immunized B6 mice, whereas in the presence of c-di-AMP, both WT and IFN-αβR-deficient OT-I cells had significantly reduced populations indicating that type I IFNs were not directly blocking T cell priming (**Fig. 8A and 8B**). Inhibition of T cell expansion by c-di-AMP was rescued in IFN-αβR- and STING-deficient mice further indicating that type I IFN-mediated suppression of immunity is not T cell intrinsic.

## Discussion

The results of this study show that the STING signaling pathway is not required to elicit CMI to *L. monocytogenes*. In fact, the absence of STING or IRF3 and IRF7 led to a higher number of antigen-specific CD8^+^ T cells and increased levels of protective immunity. Mice lacking both MyD88 and STING were also protected upon secondary challenge, indicating that not only is STING dispensable for the generation of a protective response to *L. monocytogenes*, it does not act in a redundant fashion with the TLR-MyD88 signaling pathway. Conversely, when STING activity was enhanced either by administering c-di-AMP during immunization or using a bacterial mutant that secretes elevated levels of c-di-AMP, mice failed to immunize and had decreased numbers of antigen-specific T cells following reinfection. This suppressive effect was largely due to the induction of type I IFNs since IFN-αβR-deficient mice immunized with either ActA^−^Lm-OVA in the presence of c-di-AMP or the *tetR* mutant were protected. Collectively, these findings suggest that *L. monocytogenes*-induced STING activation reduces the host adaptive immune response by induction of type I IFN.

The mechanism of type I IFN-mediated inhibition of T cell priming remains unclear. We found that both WT and IFN-αβR-deficient CD8^+^ T cells had significantly reduced expansion in the presence of c-di-AMP, suggesting that c-di-AMP-mediated suppression of lymphocyte priming is T cell extrinsic. One possibility is that the uptake of type I IFN-induced apoptotic cells by macrophages results in the release of the immunosuppressive cytokine IL-10 [35]. However, mice administered c-di-AMP did not have detectable levels of IL-10 in the serum. Furthermore, similar to B6 mice, IL-10-deficient mice immunized in the presence of c-di-AMP were unable to elicit OVA-specific CD8^+^ T cells following challenge (**data not shown**). Thus, IL-10 is not the downstream effector of STING/type I IFN-mediated suppression of adaptive immunity in our model of protective immunity. Other mechanisms including indoleamine 2,3-dioxygenase (IDO)-mediated T cell suppression, which is upregulated by type I IFN [36], may play a role in c-di-AMP-mediated inhibition of immunity. Indeed, Huang *et al.* found that splenic DCs from mice treated with DNA nanoparticles suppress *ex vivo* T cell proliferation in a STING- and IDO-dependent manner [37].

While spleens from immunized STING-deficient mice contained fewer bacteria than B6 mice, bacterial clearance by immunized IRF3/7- and IFN-αβR-deficient mice was even higher. One possibility is that there are STING-independent sources of type I IFNs in response to *L. monocytogenes*. Supporting this hypothesis, we observed that MyD88/STING-deficient macrophages produced low levels of IFN-β eight hours post infection. Recent studies found that RIG-I-deficient cells had reduced IFN-β secretion in response to *L. monocytogenes* infection suggesting that RNA from *L. monocytogenes* can be detected by the host [38], [39]. Supporting this hypothesis, we found that immunized mice lacking MAVS, the signaling adaptor for RIG-I, were more protected than control mice upon reinfection (**data not shown**). Thus, RIG-I-dependent, STING-independent induction of type I IFNs via IRF3 and IRF7 may also contribute to the inhibition of protective immunity. Another possibility is that since naïve IRF3/7- and IFN-αβR-deficient mice exhibit heightened bacterial clearance compared to both naïve B6 and STING-deficient mice, innate immune factors are also likely restricting reinfection. In fact, IFN-αβR-deficient mice have higher neutrophil recruitment in response to *L. monocytogenes* infection [40]. Thus, careful examination of each mouse strain will be necessary to determine the extent of the contribution of innate versus adaptive immune mechanisms in protective immunity.

Type I IFN-mediated inhibition of immunity is unlikely specific to STING activation. Mice immunized in the presence of the poly(I∶C), which induces type I IFNs independently of STING, also lost the ability to restrict bacterial growth following challenge. Several studies have shown that administering poly(I∶C) prior to a protein antigen inhibits clonal expansion of antigen-specific CD8^+^ T cells, supporting our findings that systemic type I IFN-induced inflammation reduces T cell priming [41], [42].

The importance of type I IFNs during bacterial infection is less understood than for viruses [43]. For example, IFN-β is the highest upregulated gene following *L. monocytogenes* cytosolic invasion, yet mice lacking the IFN-αβR are more resistant to acute infection, suggesting that type I IFNs may promote pathogenesis [35], [44], [45]. However, strains of *L. monocytogenes* that secrete elevated levels of c-di-AMP and induce higher levels of type I IFNs, are not hypervirulent [46], but induce considerably less T cell immunity as shown in this study. Thus it is possible that secretion of c-di-AMP and consequent type I IFN production may play a role in *L. monocytogenes* pathogenesis by suppressing the development of adaptive immunity. Although *L. monocytogenes* generally causes acute infections, recent studies have found that type I IFNs promote chronic infections with LCMV by suppressing cell-mediated mechanisms of viral control [47], [48]. In the case of *Mycobacterium tuberculosis*, mice lacking IRF3 are more resistant to infection with *M. tuberculosis* suggesting that IRF3 activation is detrimental to host clearance [49]. In humans, IFN-α treatment leads to higher incidences of TB reactivation [50]. Furthermore, active TB patients exhibit an increase in type I IFN-inducible transcripts in the blood, which correlated with disease severity [51]. Therefore, type I IFNs may exacerbate or maintain secondary or long-term chronic infections. Interestingly, human STING often contains polymorphisms that makes it resistant to bacterial but not host derived CDNs [52].

STING-mediated suppression of protective immunity was not solely due to type I IFNs. Although protective immunity in *tetR*ActA^−^Lm-OVA-immunized mice was rescued in the absence of IRF3/7 and the IFN-αβR, mice immunized in the presence of c-di-AMP exhibited type I IFN-independent suppression. Since STING activates NF-κB as well as IRF3, it is possible that NF-κB-dependent inflammation also plays a role in restricting immunity. We believe that administering c-di-AMP activates STING more robustly compared to infection with the *tetR*ActA^−^Lm strain and thus inhibition of immunity by type I IFN-independent inflammation would become more apparent.

The results of this and other studies suggest an inverse relationship between the extent of inflammation and the development of adaptive immunity. For example, IL-12-deficient mice immunized with *L. monocytogenes* develop higher numbers of CD8^+^ memory T cells and are more resistant to reinfection [53]. In previous studies, we found that a *L. monocytogenes* strain engineered to activate the inflammasome, and consequently induce high levels of IL-1β secretion, was a poor inducer of adaptive immunity [54]. Furthermore, co-administration of heat-killed or LLO-deficient *L. monocytogenes* blocked immunity to WT bacteria in a MyD88-dependent manner [55]. Thus, activation of three distinct signaling pathways, STING, MyD88, and caspase-1 all resulted in the inhibition of the development of adaptive immunity. Therefore, there appears to be a dichotomy between innate immune pathways that are necessary for survival (for example, MyD88 for *L. monocytogenes* and type I IFN for viruses), and those that lead to adaptive immunity. In fact, our data and work from others suggest that lack of inflammation represents an ideal environment for the generation of memory T cells [31], [56]. Indeed, mice deficient for both MyD88 and STING are fully immunized by *L. monocytogenes* even though there was a significant reduction of dendritic and T cell activation and cytokine production following immunization.

Considering that the innate immune response is believed to be required for the initiation of adaptive immunity, we were surprised that MyD88/STING-deficient mice immunized with *L. monocytogenes* were protected after reinfection. This raises the question, which innate immune signaling pathways contribute to the initiation of T cell priming to *L. monocytogenes*? Previous work from our group found that NOD2 detects cytosolic *L. monocytogenes* [5]. However, immunized MyD88/NOD1/2-deficient mice clear bacteria upon secondary lethal challenge (**data not shown**), suggesting that protective immunity is not due to redundancy between MyD88 and NOD-like signaling pathways. Future studies to identify which innate immune detection pathways are required for *L. monocytogenes*-mediated CMI would provide a greater understanding of how pathogens and adjuvants elicit protective immunity, knowledge that can be used for the development and improvement of vaccines.

## Materials and Methods

### Ethics statement

This study was carried out in strict accordance with the recommendations in the Guide for the Care and Use of Laboratory Animals of the National Institutes of Health. All protocols were reviewed and approved by the Animal Care and Use Committee at the University of California, Berkeley (MAUP# R235-0813B).

### Mouse strains

C57BL/6 mice were purchased from The Jackson Laboratory. *Goldenticket* (*Gt*) mice were generated from an ENU mutagenesis screen. *Gt* mice contain a single nucleotide mutation in STING resulting in the absence of the STING protein [17]. All mice were in the C57BL/6 genetic background. *Gt*, *MyD88^−/−^*, *MyD88^−/−^Gt*, *IRF3^−/−^*, *IRF3/7^−/−^* and *IFNAR1^−/−^* mice were bred in our facilities. *GFP^+/+^ OT-I^+/+^ RAG2^−/−^* and *IFNAR1^−/−^ OT-I^+/+^ Ly5.1^+/+^ RAG2^−/−^* mice were generously provided by Ellen Robey.

### Bacterial strains and culture

All *L. monocytogenes* strains were in the 10403S background. ActA^−^Lm-OVA (Δ*actA*Δ*inlB*) (DP-L6014) [57], WT Lm-OVA (DP-L6018) and LLO^−^Lm-OVA (Δ*hly*) (DP-L6017) [21] *L. monocytogenes* were previously described. For *tetR*ActA^−^Lm-OVA (DP-L6015), the *tetR*::Tn*917* transposon [14] was transduced into ActA^−^Lm-OVA. *L. monocytogenes* were grown in brain heart infusion (BHI) media at 30°C overnight without shaking to stationary phase. For *in vitro* infections, *L. monocytogenes* was washed 3× in PBS. For *in vivo* infections, *L. monocytogenes* was diluted in BHI at 37°C shaking for ∼2 hours until they reached an OD_600_ 0.4–0.6.

### *In vivo* infections

Eight to 12 week old sex-matched mice were infected intravenously with 10^3^ CFU (unless otherwise indicated) of *L. monocytogenes* diluted in phosphate buffered saline (PBS) in a total volume of 200 µl. For acute infections and primary immunization studies, mice were sacrificed at 1, 2 and 3 or 7 days post infection, respectively. For challenge studies, mice immunized 30–38 days prior were infected with 2×10^5^ CFU of WT Lm-OVA. Where indicated, mice were administered either 50 µg or 100 µg of c-di-AMP or 50 µg of poly(I∶C) (InvivoGen) with the bacterial inoculum. Three days later, spleens and livers were homogenized in 0.1% IGEPAL CA-630 (Sigma) and plated on LB-strep plates to enumerate CFU. For analysis of CD8^+^ T cell responses, spleens were divided and weighed. For splenic dendritic and T cell activation, mice were immunized with 10^5^ CFU of ActA^−^Lm-OVA. A higher dose was used to allow for the easy detection of activated cells.

For OT-I cells, splenocytes from *GFP^+/+^ OT-I^+/+^ RAG2^−/−^* and *IFNAR1^−/−^ OT-I^+/+^ Ly5.1^+/+^ RAG2^−/−^* mice were isolated and washed 3× with PBS. Percent of CD8^+^ OT-I cells was determined by flow cytometry. 2×10^4^ of each cell type was injected intravenously into each mouse (4×10^4^ total cells/mouse) 1 day prior to immunization with *L. monocytogenes*.

C-di-AMP was generated by Josh Woodward as previously described [58]. Purified c-di-AMP was resuspended in tissue culture grade PBS. LPS was removed from the prepared c-di-AMP using Detoxi-gel endotoxin removing gel (Pierce) according to the manufacturers instructions. Endotoxin content was measured using the Toxinsensor Chromogenic LAL Endotoxin assay kit (Genescript). The nucleotide solution was passed through the Detoxi-gel until endotoxin levels were <0.0125 EU/ml. Nucleotide was then diluted to 500 µg/ml.

### Dendritic cell and T cell analysis

For analysis of T cell responses, spleen halves were dissociated and filtered through a 70 µm cell strainer. Red blood cells were lysed with Red Blood Cell Lysing Buffer (Sigma). To determine OVA-specific cells, splenocytes were stained with anti-mouse CD8, CD44, CD62L and a K^b^/OVA_257–264_ tetramer. Representative FACs plots are gated on CD8^+^ cells and values show the median percentage of K^b^/OVA_257–264_ tetramer^+^ CD44^+^ within the CD8^+^ cell population ± SEM. For peptide stimulation assays, splenocytes were stimulated for 5 hours with 2 µM OVA_257–264_ or LLO_190–201_ peptide in the presence of GolgiPlug (BD Biosciences). Cells were surface stained with anti-mouse CD8 and CD4, fixed and permeabilized using Cytofix/Cytoperm (BD Biosciences), and stained for intracellular anti-mouse IFN-γ, TNF-α and IL-2. For splenic dendritic cells and T cells, splenocytes were stained with anti-mouse CD11b, CD11c, CD86 and CD40 or anti-mouse CD8, CD4 and CD69, respectively. Flurophore-conjugated antibodies were purchased from eBioscience. Samples were acquired using an LSRII flow cytometer (BD Biosciences) and analyzed using FlowJo software (Tree Star).

### Quantitative PCR

BMMs were generated as previously described [59]. In a 6-well plate, 2×10^6^ BMMs were either infected with WT Lm at a multiplicity of infection (MOI) of 2 bacteria per cell or stimulated with 10 µM c-di-AMP. At 30 minutes post infection, gentamicin was added for a final concentration of 50 µg/ml. At 4 and 8 hours post infection, cells were harvested and RNA was purified using the RNAqueous kit (Ambion). RNA was then DNase treated, processed and analyzed as previously described [5].

### Bone marrow-derived dendritic cells

Bone marrow from femurs was plated in media containing 20 ng/ml recombinant murine GMCSF (ProSpec) at a density of 5×10^5^ cells/ml in a 24-well plate. At days 2, 4 and 5 media was replaced with fresh media containing 20 ng/ml GMCSF and cells were harvested on day 6. BMDCs were plated at 3×10^5^ cells/well in 48-well plates. Cells were incubated with either 10 µM c-di-AMP, 100 ng/ml lipopolysaccharide (LPS), or 20 µg/ml poly(I∶C) (InvivoGen). After 24 hours, supernatant was assayed for IFN-β using ISRE-L929 cells as previously described [14], or for MCP-1, IL-12p40 (BD OptEIA kit, BD Biosciences) and IL-6 (eBioscience) by ELISA.

### Serum cytokines

Serum cytokines were measured using the CBA Mouse Inflammation Kit (BD Biosciences) and analyzed on the LSRII flow cytometer.

### Statistical analysis

A two-tailed, Mann-Whitney *U* test was used to analyze the significance of differences in the means between groups. Significance is indicated as * p<0.05, ** p<0.005, *** p<0.0005 or ns = not significant.

## Supporting Information

Figure S1 **Low immunization doses of avirulent** ***L. monocytogenes*** **protect against secondary lethal challenge.** **A**. B6 mice, and **B**. B6 and *Gt* mice were immunized intravenously with 10^7^, 10^6^, 10^5^, 10^4^ or 10^3^ CFU ActA^−^Lm-OVA (Δ*actA)* (open circles) or administered PBS (closed circles). Mice were challenged 30–38 days following immunization with 2×10^5^ CFU WT Lm-OVA and 3 days later CFU were enumerated in the spleens or livers of each mouse. The dashed line represents the limit of detection. Data are presented as cumulative results from 1–5 independent experiments (***p<0.0005).
(EPS)

Figure S2 **MyD88/STING-deficient mice have a defect in** ***L. monocytogenes*** **recognition and are more susceptible to acute infection.** **A**. BMMs from B6 (solid bars), *Gt* (open bars), *MyD88^−/−^* (lined bars) and *MyD88^−/−^Gt* (dotted bars) mice were infected with WT Lm (MOI 2) or incubated with 10 µM c-di-AMP. RNA was harvested 4 or 8 hours later and indicated cytokine transcripts were measured relative to those of β-actin transcripts. Data are presented as fold over uninfected BMMs and represent the mean ± SEM from 2 independent experiments. **B**. B6 (solid circles), *MyD88^−/−^* and *MyD88^−/−^Gt* (open circles) mice were infected with 10^3^ CFU WT Lm and bacterial numbers in the spleens and livers were enumerated at days 1, 2 and 3 post infection. An **X** marks each mouse that succumbed to infection prior to the conclusion of experiment. Data are presented as cumulative results from 3–4 independent experiments (ND = not detectable, ns = not significant, *p<0.05, **p<0.005, ***p<0.0005).
(EPS)

Figure S3 **C-di-AMP activates dendritic cells and T cells in a STING-dependent manner** ***in vitro*** **.** **A**. BMDCs generated from B6 (solid bars) and *Gt* (open bars) mice were incubated with either 10 µM c-di-AMP, 20 µg/ml polyI∶C, 100 ng/ml LPS or PBS for 24 hours. IFN-β was determined by ISRE bioassay. MCP-1, IL-12p40 and IL-6 were determined by ELISA. **B**. BMDCs from **A** were stained with anti-mouse CD86 (top panels) and CD40 (bottom panels) and analyzed by flow cytometry. Histograms show unstimulated cells (shaded), 10 µM c-di-AMP (solid line) and 20 µg/mL polyI∶C (dashed line). Data are quantified as the fold increase of median fluorescence intensity over uninfected cells and presented as the mean ± SEM from 4 independent experiments.
(EPS)

Figure S4 **Enhanced STING activation during immunization inhibits expansion of total number of CD8^+^ T cells upon** ***L. monocytogenes*** **reinfection.** **A**. B6, *Gt* or *IRF3/7^−/−^* mice were immunized with either 10^3^ CFU Δ*actA* in the presence (open triangles) or absence (open circles) of 100 µg c-di-AMP or **B**. B6 mice were immunized with either 10^3^ CFU Δ*actA* (open circles) or *tetR*ActA^−^Lm-OVA (*tetR*Δ*actA*) (open triangles). Naive controls were administered sterile PBS (closed circles). Mice were challenged 30–38 days post immunization with 2×10^5^ CFU WT Lm-OVA and 3 days later splenocytes were isolated and stained with anti-mouse CD8 and analyzed by flow cytometry. Quantitative analysis shows the total number of CD8^+^ T cells/spleen. Data are presented as the cumulative results from 2–3 (**A**) and 3 (**B**) independent experiments (ns = not significant, *p<0.05, **p<0.005, ***p<0.0005).
(EPS)

Figure S5 **Administration of poly(I∶C) during immunization inhibits protective immunity upon** ***L. monocytogenes*** **reinfection.** B6 mice were immunized with either 10^3^ CFU Δ*actA* in the presence (open triangles) or absence (open circles) of 50 µg poly(I∶C). Naive controls were administered sterile PBS (closed circles). Mice were challenged 30–38 days post immunization with 2×10^5^ CFU WT Lm-OVA and 3 days later CFU were enumerated in spleens and livers. The dashed line represents limit of detection. Data are presented as the cumulative results from 2 independent experiments (**p<0.005).
(EPS)

## References

[1] Harty JT, Tvinnereim AR, White DW (2000) CD8+ T cell effector mechanisms in resistance to infection. Annual review of immunology 18: 275–308. 10.1146/annurev.immunol.18.1.275 10837060

[2] Pamer EG (2004) Immune responses to Listeria monocytogenes. Nat Rev Immunol 4: 812–823. 15459672 10.1038/nri1461

[3] Schenten D, Medzhitov R (2011) The control of adaptive immune responses by the innate immune system. Advances in immunology 109: 87–124. 21569913 10.1016/B978-0-12-387664-5.00003-0

[4] Witte CE, Archer KA, Rae CS, Sauer JD, Woodward JJ, et al (2012) Innate immune pathways triggered by Listeria monocytogenes and their role in the induction of cell-mediated immunity. Advances in immunology 113: 135–156. 22244582 10.1016/B978-0-12-394590-7.00002-6

[5] Leber JH, Crimmins GT, Raghavan S, Meyer-Morse NP, Cox JS, et al (2008) Distinct TLR- and NLR-mediated transcriptional responses to an intracellular pathogen. PLoS Pathog 4: e6. 18193943 10.1371/journal.ppat.0040006 PMC2186359

[6] Cossart P, Toledo-Arana A (2008) Listeria monocytogenes, a unique model in infection biology: an overview. Microbes and infection/Institut Pasteur 10: 1041–1050. 10.1016/j.micinf.2008.07.043 18775788

[7] O'Riordan M, Yi CH, Gonzales R, Lee KD, Portnoy DA (2002) Innate recognition of bacteria by a macrophage cytosolic surveillance pathway. Proc Natl Acad Sci U S A 99: 13861–13866. 12359878 10.1073/pnas.202476699 PMC129788

[8] Barber GN (2011) Innate immune DNA sensing pathways: STING, AIMII and the regulation of interferon production and inflammatory responses. Current opinion in immunology 23: 10–20. 21239155 10.1016/j.coi.2010.12.015 PMC3881186

[9] Barker JR, Koestler BJ, Carpenter VK, Burdette DL, Waters CM, et al (2013) STING-dependent recognition of cyclic di-AMP mediates type I interferon responses during Chlamydia trachomatis infection. mBio 4: e00018–00013. 23631912 10.1128/mBio.00018-13 PMC3663186

[10] Zhang X, Shi H, Wu J, Sun L, Chen C, et al (2013) Cyclic GMP-AMP Containing Mixed Phosphodiester Linkages Is An Endogenous High-Affinity Ligand for STING. Molecular cell 51: 226–235. 23747010 10.1016/j.molcel.2013.05.022 PMC3808999

[11] Burdette DL, Monroe KM, Sotelo-Troha K, Iwig JS, Eckert B, et al (2011) STING is a direct innate immune sensor of cyclic di-GMP. Nature 478: 515–518. 21947006 10.1038/nature10429 PMC3203314

[12] Ishikawa H, Ma Z, Barber GN (2009) STING regulates intracellular DNA-mediated, type I interferon-dependent innate immunity. Nature 461: 788–792. 19776740 10.1038/nature08476 PMC4664154

[13] McWhirter SM, Barbalat R, Monroe KM, Fontana MF, Hyodo M, et al (2009) A host type I interferon response is induced by cytosolic sensing of the bacterial second messenger cyclic-di-GMP. J Exp Med 206: 1899–1911. 19652017 10.1084/jem.20082874 PMC2737161

[14] Crimmins GT, Herskovits AA, Rehder K, Sivick KE, Lauer P, et al (2008) Listeria monocytogenes multidrug resistance transporters activate a cytosolic surveillance pathway of innate immunity. Proc Natl Acad Sci U S A 105: 10191–10196. 18632558 10.1073/pnas.0804170105 PMC2481368

[15] Woodward JJ, Iavarone AT, Portnoy DA (2010) c-di-AMP secreted by intracellular Listeria monocytogenes activates a host type I interferon response. Science 328: 1703–1705. 20508090 10.1126/science.1189801 PMC3156580

[16] Jin L, Hill KK, Filak H, Mogan J, Knowles H, et al (2011) MPYS is required for IFN response factor 3 activation and type I IFN production in the response of cultured phagocytes to bacterial second messengers cyclic-di-AMP and cyclic-di-GMP. Journal of immunology 187: 2595–2601. 10.4049/jimmunol.1100088 PMC3159690 21813776

[17] Sauer JD, Sotelo-Troha K, von Moltke J, Monroe KM, Rae CS, et al (2011) The N-Ethyl-N-Nitrosourea-Induced Goldenticket Mouse Mutant Reveals an Essential Function of Sting in the In Vivo Interferon Response to Listeria monocytogenes and Cyclic Dinucleotides. Infect Immun 79: 688–694. 21098106 10.1128/IAI.00999-10 PMC3028833

[18] Ebensen T, Libanova R, Schulze K, Yevsa T, Morr M, et al (2011) Bis-(3′,5′)-cyclic dimeric adenosine monophosphate: Strong Th1/Th2/Th17 promoting mucosal adjuvant. Vaccine 29: 5210–5220. 21619907 10.1016/j.vaccine.2011.05.026

[19] Ebensen T, Schulze K, Riese P, Link C, Morr M, et al (2007) The bacterial second messenger cyclic diGMP exhibits potent adjuvant properties. Vaccine 25: 1464–1469. 17187906 10.1016/j.vaccine.2006.10.033

[20] Karaolis DK, Means TK, Yang D, Takahashi M, Yoshimura T, et al (2007) Bacterial c-di-GMP is an immunostimulatory molecule. J Immunol 178: 2171–2181. 17277122 10.4049/jimmunol.178.4.2171

[21] Bahjat KS, Liu W, Lemmens EE, Schoenberger SP, Portnoy DA, et al (2006) Cytosolic entry controls CD8+-T-cell potency during bacterial infection. Infect Immun 74: 6387–6397. 16954391 10.1128/IAI.01088-06 PMC1695486

[22] Berche P, Gaillard JL, Sansonetti PJ (1987) Intracellular growth of Listeria monocytogenes as a prerequisite for in vivo induction of T cell-mediated immunity. J Immunol 138: 2266–2271. 3104455

[23] von Koenig CH, Finger H, Hof H (1982) Failure of killed Listeria monocytogenes vaccine to produce protective immunity. Nature 297: 233–234. 6176874 10.1038/297233a0

[24] Harty JT, Bevan MJ (1995) Specific immunity to Listeria monocytogenes in the absence of IFN gamma. Immunity 3: 109–117. 7621071 10.1016/1074-7613(95)90163-9

[25] Edelson BT, Unanue ER (2002) MyD88-dependent but Toll-like receptor 2-independent innate immunity to Listeria: no role for either in macrophage listericidal activity. Journal of immunology 169: 3869–3875. 10.4049/jimmunol.169.7.3869 12244184

[26] Kursar M, Mittrucker HW, Koch M, Kohler A, Herma M, et al (2004) Protective T cell response against intracellular pathogens in the absence of Toll-like receptor signaling via myeloid differentiation factor 88. International immunology 16: 415–421. 14978015 10.1093/intimm/dxh047

[27] Seki E, Tsutsui H, Tsuji NM, Hayashi N, Adachi K, et al (2002) Critical roles of myeloid differentiation factor 88-dependent proinflammatory cytokine release in early phase clearance of Listeria monocytogenes in mice. Journal of immunology 169: 3863–3868. 10.4049/jimmunol.169.7.3863 12244183

[28] Way SS, Kollmann TR, Hajjar AM, Wilson CB (2003) Cutting edge: protective cell-mediated immunity to Listeria monocytogenes in the absence of myeloid differentiation factor 88. J Immunol 171: 533–537. 12847214 10.4049/jimmunol.171.2.533

[29] Chen H, Sun H, You F, Sun W, Zhou X, et al (2011) Activation of STAT6 by STING is critical for antiviral innate immunity. Cell 147: 436–446. 22000020 10.1016/j.cell.2011.09.022

[30] Ishikawa H, Barber GN (2008) STING is an endoplasmic reticulum adaptor that facilitates innate immune signalling. Nature 455: 674–678. 18724357 10.1038/nature07317 PMC2804933

[31] Condotta SA, Richer MJ, Badovinac VP, Harty JT (2012) Probing CD8 T cell responses with Listeria monocytogenes infection. Advances in immunology 113: 51–80. 22244579 10.1016/B978-0-12-394590-7.00005-1

[32] Stockinger S, Kastner R, Kernbauer E, Pilz A, Westermayer S, et al (2009) Characterization of the interferon-producing cell in mice infected with Listeria monocytogenes. PLoS pathogens 5: e1000355. 19325882 10.1371/journal.ppat.1000355 PMC2654726

[33] Keppler SJ, Rosenits K, Koegl T, Vucikuja S, Aichele P (2012) Signal 3 cytokines as modulators of primary immune responses during infections: the interplay of type I IFN and IL-12 in CD8 T cell responses. PLoS One 7: e40865. 22815848 10.1371/journal.pone.0040865 PMC3398954

[34] Thompson LJ, Kolumam GA, Thomas S, Murali-Krishna K (2006) Innate inflammatory signals induced by various pathogens differentially dictate the IFN-I dependence of CD8 T cells for clonal expansion and memory formation. J Immunol 177: 1746–1754. 16849484 10.4049/jimmunol.177.3.1746

[35] Carrero JA, Calderon B, Unanue ER (2004) Type I interferon sensitizes lymphocytes to apoptosis and reduces resistance to Listeria infection. J Exp Med 200: 535–540. 15302900 10.1084/jem.20040769 PMC2211931

[36] Huang L, Lemos HP, Li L, Li M, Chandler PR, et al (2012) Engineering DNA nanoparticles as immunomodulatory reagents that activate regulatory T cells. Journal of immunology 188: 4913–4920. 10.4049/jimmunol.1103668 PMC3349160 22516958

[37] Huang L, Li L, Lemos H, Chandler PR, Pacholczyk G, et al (2013) Cutting Edge: DNA Sensing via the STING Adaptor in Myeloid Dendritic Cells Induces Potent Tolerogenic Responses. Journal of immunology 191: 3509–13. 10.4049/jimmunol.1301419 PMC3788571 23986532

[38] Abdullah Z, Schlee M, Roth S, Mraheil MA, Barchet W, et al (2012) RIG-I detects infection with live Listeria by sensing secreted bacterial nucleic acids. The EMBO journal 31: 4153–4164. 23064150 10.1038/emboj.2012.274 PMC3492734

[39] Hagmann CA, Herzner AM, Abdullah Z, Zillinger T, Jakobs C, et al (2013) RIG-I Detects Triphosphorylated RNA of Listeria monocytogenes during Infection in Non-Immune Cells. PLoS One 8: e62872. 23653683 10.1371/journal.pone.0062872 PMC3639904

[40] Brzoza-Lewis KL, Hoth JJ, Hiltbold EM (2012) Type I interferon signaling regulates the composition of inflammatory infiltrates upon infection with Listeria monocytogenes. Cellular immunology 273: 41–51. 22212606 10.1016/j.cellimm.2011.11.008 PMC3264831

[41] Marshall HD, Urban SL, Welsh RM (2011) Virus-induced transient immune suppression and the inhibition of T cell proliferation by type I interferon. Journal of virology 85: 5929–5939. 21471240 10.1128/JVI.02516-10 PMC3126308

[42] Ngoi SM, St Rose MC, Menoret AM, Smith DE, Tovey MG, et al (2012) Presensitizing with a Toll-like receptor 3 ligand impairs CD8 T-cell effector differentiation and IL-33 responsiveness. Proceedings of the National Academy of Sciences of the United States of America 109: 10486–10491. 22689946 10.1073/pnas.1202607109 PMC3387033

[43] Monroe KM, McWhirter SM, Vance RE (2010) Induction of type I interferons by bacteria. Cellular microbiology 12: 881–890. 20482555 10.1111/j.1462-5822.2010.01478.x PMC2897911

[44] Auerbuch V, Brockstedt DG, Meyer-Morse N, O'Riordan M, Portnoy DA (2004) Mice lacking the type I interferon receptor are resistant to Listeria monocytogenes. J Exp Med 200: 527–533. 15302899 10.1084/jem.20040976 PMC2211930

[45] O'Connell RM, Saha SK, Vaidya SA, Bruhn KW, Miranda GA, et al (2004) Type I interferon production enhances susceptibility to Listeria monocytogenes infection. J Exp Med 200: 437–445. 15302901 10.1084/jem.20040712 PMC2211937

[46] Schwartz KT, Carleton JD, Quillin SJ, Rollins SD, Portnoy DA, et al (2012) Hyperinduction of host beta interferon by a Listeria monocytogenes strain naturally overexpressing the multidrug efflux pump MdrT. Infection and immunity 80: 1537–1545. 22290148 10.1128/IAI.06286-11 PMC3318417

[47] Wilson EB, Yamada DH, Elsaesser H, Herskovitz J, Deng J, et al (2013) Blockade of chronic type I interferon signaling to control persistent LCMV infection. Science 340: 202–207. 23580528 10.1126/science.1235208 PMC3704950

[48] Teijaro JR, Ng C, Lee AM, Sullivan BM, Sheehan KC, et al (2013) Persistent LCMV infection is controlled by blockade of type I interferon signaling. Science 340: 207–211. 23580529 10.1126/science.1235214 PMC3640797

[49] Manzanillo PS, Shiloh MU, Portnoy DA, Cox JS (2012) Mycobacterium tuberculosis activates the DNA-dependent cytosolic surveillance pathway within macrophages. Cell host & microbe 11: 469–480. 22607800 10.1016/j.chom.2012.03.007 PMC3662372

[50] Telesca C, Angelico M, Piccolo P, Nosotti L, Morrone A, et al (2007) Interferon-alpha treatment of hepatitis D induces tuberculosis exacerbation in an immigrant. The Journal of infection 54: e223–226. 17307255 10.1016/j.jinf.2006.12.009

[51] Berry MP, Graham CM, McNab FW, Xu Z, Bloch SA, et al (2010) An interferon-inducible neutrophil-driven blood transcriptional signature in human tuberculosis. Nature 466: 973–977. 20725040 10.1038/nature09247 PMC3492754

[52] Diner EJ, Burdette DL, Wilson SC, Monroe KM, Kellenberger CA, et al (2013) The Innate Immune DNA Sensor cGAS Produces a Noncanonical Cyclic Dinucleotide that Activates Human STING. Cell reports 3: 1355–1361. 23707065 10.1016/j.celrep.2013.05.009 PMC3706192

[53] Pearce EL, Shen H (2007) Generation of CD8 T cell memory is regulated by IL-12. Journal of immunology 179: 2074–2081. 10.4049/jimmunol.179.4.2074 17675465

[54] Sauer JD, Pereyre S, Archer KA, Burke TP, Hanson B, et al (2011) Listeria monocytogenes engineered to activate the Nlrc4 inflammasome are severely attenuated and are poor inducers of protective immunity. Proceedings of the National Academy of Sciences of the United States of America 108: 12419–12424. 21746921 10.1073/pnas.1019041108 PMC3145703

[55] Bahjat KS, Meyer-Morse N, Lemmens EE, Shugart JA, Dubensky TW, et al (2009) Suppression of cell-mediated immunity following recognition of phagosome-confined bacteria. PLoS pathogens 5: e1000568. 19730694 10.1371/journal.ppat.1000568 PMC2731223

[56] Badovinac VP, Porter BB, Harty JT (2004) CD8+ T cell contraction is controlled by early inflammation. Nature immunology 5: 809–817. 15247915 10.1038/ni1098

[57] Brockstedt DG, Giedlin MA, Leong ML, Bahjat KS, Gao Y, et al (2004) Listeria-based cancer vaccines that segregate immunogenicity from toxicity. Proc Natl Acad Sci U S A 101: 13832–13837. 15365184 10.1073/pnas.0406035101 PMC518841

[58] Witte CE, Whiteley AT, Burke TP, Sauer JD, Portnoy DA, et al (2013) Cyclic di-AMP Is Critical for Listeria monocytogenes Growth, Cell Wall Homeostasis, and Establishment of Infection. mBio 4: e00282–13. 23716572 10.1128/mBio.00282-13 PMC3663569

[59] Portnoy DA, Jacks PS, Hinrichs DJ (1988) Role of hemolysin for the intracellular growth of Listeria monocytogenes. J Exp Med 167: 1459–1471. 2833557 10.1084/jem.167.4.1459 PMC2188911

